# Motivation and goal-pursuit for injury prevention training in amateur football coaches: a cross-sectional study using the Health Action Process Approach

**DOI:** 10.1136/ip-2023-044978

**Published:** 2023-09-11

**Authors:** Hanna Lindblom, Martin Hägglund

**Affiliations:** 1 Department of Health, Medicine and Caring Sciences, Unit of Physiotherapy, Linköping University, Linköping, Sweden; 2 Department of Health, Medicine and Caring Sciences, Sport Without Injury ProgrammE (SWIPE), Linköping University, Linköping, Sweden

**Keywords:** Behavior Change, Training, Implementation / Translation, Sports / Leisure Facility

## Abstract

**Background:**

Adoption of injury prevention exercise programmes (IPEPs) in team sports is contingent on behaviour change among coaches. The aim was to study motivation and goal-pursuit in IPEP use among coaches of amateur football players.

**Methods:**

A cross-sectional study using web-based questionnaires was administered to coaches in one Swedish regional football district. The study was carried out one season after dissemination of the IPEP *Knee Control+*. The questionnaire was based on the Health Action Process Approach and covered perceptions and beliefs about using *Knee Control+*. Questions were rated on 1–7 Likert scales.

**Results:**

440 coaches participated (response rate 32%). Coaches were neutral about injury risks (median 4–5) and knowledge about preventing injuries (median 5) but had positive outcome expectancies of preventive training (median 6). Coaches who had used an IPEP perceived they had more knowledge about preventing injuries than non-users (median 5 vs 4, small effect size *d*=0.43). Coaches who used *Knee Control+* were positive about their practical ability to use it (median 6) and had high intention to prioritise continuous use (median 7). Highly adherent coaches to higher extent believed that specific training may prevent injuries and had plans for how to instruct the players and how to work around barriers compared with low adherent coaches.

**Conclusion:**

Coaches need more knowledge and support on IPEP usage and how to structure training. Coaches who had adopted *Knee Control+* had high belief in their abilities but may need constructive plans on how to use the programme and to overcome barriers.

**WHAT IS ALREADY KNOWN ON THIS TOPIC**

- Injury prevention exercise programmes (IPEPs) are efficacious in preventing injuries in team sports.
- Coaches often modify IPEPs’ content or dosage and there is a need for improved implementation of IPEPs outside the controlled context of randomised controlled trials.
- In amateur sports, implementation of IPEPs is primarily the responsibility of coaches.

**WHAT THIS STUDY ADDS**

- The study covered both the motivational and volitional (goal-pursuit) phase of the Health Action Process Approach and showed neutral to high belief among coaches regarding their ability to use *Knee Control+* as well as positive outcome expectancies.
- Coaches who had used an IPEP during the season had higher belief in their knowledge about injury prevention compared with non-users.
- Coaches who had started to use *Knee Control+* during the season had positive perceptions of their ability to maintain programme use and to resume programme use after interruptions but were uncertain about plans for how to use the programme and how to cope with barriers to programme use.

**HOW THIS STUDY MIGHT AFFECT RESEARCH, PRACTICE OR POLICY**

- Our findings—with mainly neutral and some positive ratings in the motivational phase of Health Action Process Approach—suggest that while coaches have intentions to use IPEPs, they may need additional motivation and support to translate this into sustainable behaviours.
- Sustainable strategies to enhance IPEP adoption and maintained use are needed. These should be aimed at multiple stakeholders in the sport injury prevention ecological system—athletes and coaches, club management as well as regional and national governing bodies.
- Coaches may need further support to form action plans for prevention programme use and coping plans for how to deal with barriers, to improve IPEP adherence.

## Introduction

Injury prevention exercise programmes (IPEPs) are efficacious in preventing sports injuries.^1–5^ However, poor adoption and modifications of programme content or dosage^6–8^ may reduce preventive effectiveness. *Knee Control+* was developed from the *Knee Control* programme,^1 9^ and from a preliminary version called *extended Knee Control*.^10^ Primary reasons to develop *Knee Control+* were to improve programme feasibility and facilitate programme adoption and maintenance by including more exercise variations and more flexible recommendations for programme use. A recent randomised trial on *extended Knee Control* confirmed the programme’s efficacy in preventing injuries to the hamstrings, knees and ankles in youth and adult amateur football players, with 29% lower injury incidence compared with players who used a self-selected IPEP.^10^

Ideally, successful implementation of IPEPs should engage several socioecological levels, such as players, parents, coaches and football associations, but in amateur sports, implementation is primarily the responsibility of coaches. Better understanding of psychosocial factors that drive behaviour change in coaches may, thus, inform strategies to improve implementation. The Health Action Process Approach (HAPA) model is a theory of health behaviour change that can be used to describe adoption and maintenance of preventive behaviours,^11^ including use of IPEPs. HAPA distinguishes between two phases in behaviour change: a motivational phase, where intention for change is created; and a volitional (goal-pursuit) phase when intention is translated into action.^11^ In both phases, coaches’ belief in their own ability (self-efficacy) is a prominent construct.^12^ Risk perceptions, outcome expectancies and action self-efficacy are key constructs in the motivational phase, whereas action and coping planning, maintenance and recovery self-efficacy are covered in the goal-pursuit phase.^12^ Within injury prevention, HAPA has been applied in studies in football^13 14^ and rugby union.^15^ Thus far, drivers for coach behaviour change have not been evaluated outside the controlled context of intervention trials.

### Aim

The aim was to study motivation and goal-pursuit for IPEP use among coaches of amateur football players.

## Methodology

### Study design

This was a cross-sectional study with a web-based questionnaire distributed in one regional football district in Sweden (out of 24 districts) after active dissemination of *Knee Control+* during the 2021 football season. The study was approved by the Swedish Ethical Review Authority Dnr 2020–07113.

### Study population and recruitment

All football clubs and all teams registered to play in series for players 12 years or older (including the junior leagues, men 3rd–8th and women 3rd–6th senior leagues and male development series) within the regional football district were the target for dissemination of *Knee Control+*. Training frequency differs between teams and age groups, but normally a minimum of two scheduled training sessions per week are expected. Coaches for these teams received a questionnaire at the end of the season (October 2021). Contact information (e-mail addresses and telephone numbers) was collected via the clubs’ webpages during preseason. All coaches for whom contact information could be retrieved were informed about the *Knee Control+* digital programme material and received the questionnaire.

### Dissemination initiatives

In March 2021, *Knee Control+* was launched on a webpage-containing films and instructions for all exercises as well as printable programme folders in a full extensive (32 pages) and a short format (two pages) (https://liu.se/forskning/swipe/knakontroll-plus). The webpage also included a digital lecture presenting injury risks and common injuries in football (addressing HAPA injury risk perceptions), effects of IPEPs on injury risks (addressing HAPA outcome expectancies) as well as information about how to use *Knee Control+* (addressing HAPA action self-efficacy).

All clubs within the district received printed and digital programme material in March 2021, and additionally, the teams’ head coaches received information about the webpage and programme material via e-mails sent out by the research group and the regional football association. Two times during the spring season (April) and one time during the autumn season (August) coaches were invited to free digital workshops (open for everyone) on *Knee Control+* where each of the six main exercises were presented in detail regarding how to perform them, tips how to instruct players and how to overcome common barriers for IPEP use (aiming to improve HAPA action self-efficacy and maintenance self-efficacy). High interaction between researchers (HL and MH) and coaches was encouraged. In total, 306 participants attended the workshops. Since previous efficacy studies that have showed positive preventive effects had a recommended training dosage of two times per week,^1 2 10^ coaches were recommended to use *Knee Control+* during all training sessions, and at least two times per week.

### Questionnaire

**Figure 1:**
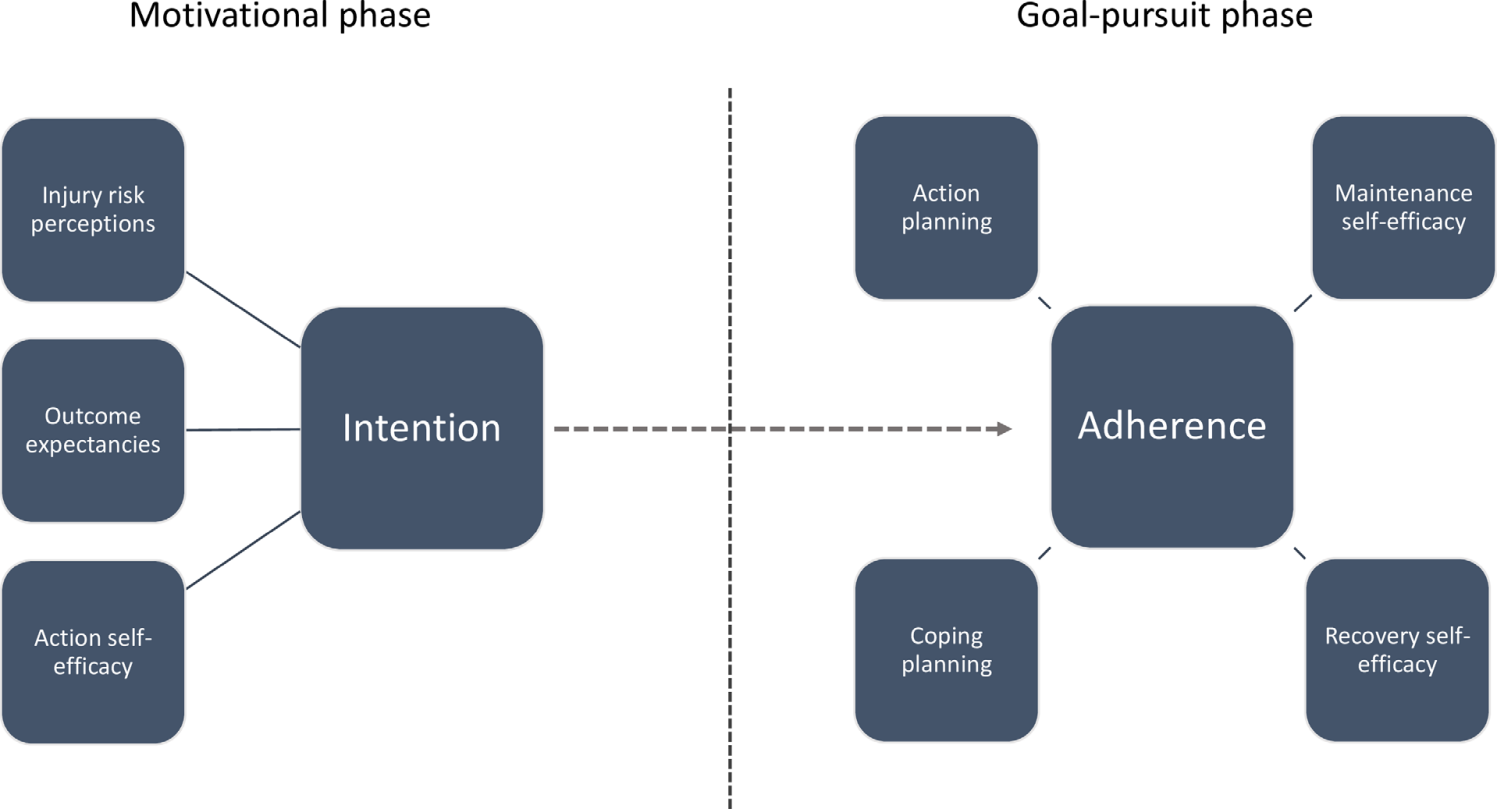
Illustration of the HAPA constructs for the motivational and goal-pursuit phases (adapted from Schwarzer).^11^ HAPA, Health Action Process Approach.

The questionnaire was distributed after the season to provide coaches with ample time to truly experience the programme, to structure action and coping plans, and experience potential difficulties related to maintenance of *Knee Control+*. The questionnaire was custom-made and covered perceptions and beliefs about using *Knee Control+*, based on the different constructs of the HAPA model (table 1, figure 1). Similar, but not identical, questions had been used in previous studies with *Knee Control*/*extended Knee Control*.^16 17^ The questions from HAPA were influenced by a study by McKay *et al*,^13^ but adapted to the Swedish context and to *Knee Control+*. Both phases of HAPA were covered for coaches who had adopted the programme, whereas coaches who had not adopted the programme only received questions related to the motivational phase. All questions were rated on a 1–7 Likert scale, where 1=negative/least beneficial to 7=positive/most beneficial and were distributed via a web-based system. No responses outside the range 1–7 were possible. In addition, coaches who had adopted *Knee Control+* listed facilitators who would aid continuous use of *Knee Control+* and barriers that may hinder continuous use of *Knee Control+* (free text responses). Up to five facilitators and five barriers could be described.

**Table 1:** Description of how each construct of the HAPA was covered in the questionnaire

HAPA construct	n questions	Coaches	Presentation
Motivational phase			
Injury risk perceptions	2	All	IPEP users vs non-users
Outcome expectancies	2	All	IPEP users vs non-users
Action self-efficacy	2	All (one question) KC+users (one question)	IPEP users vs non-users Descriptive
Intention to maintain use of *Knee Control+*	1	KC+users	Descriptive
Goal-pursuit phase			
Action planning	1	KC+users	Descriptive
Maintenance self-efficacy	1	KC+users	Descriptive
Coping planning	1	KC+users	Descriptive
Recovery self-efficacy	1	KC+users	Descriptive

All questions were rated on a 1–7 Likert scale. ‘IPEP users’ include coaches who used *Knee Control*, *Knee Control+* or the *11+*.

HAPA, Health Action Process Approach; IPEP, injury prevention exercise programme; KC+, *Knee Control+*.

All eligible coaches were targeted with the questionnaire. A questionnaire link was sent by SMS and e-mail to 1389 coaches on 5 October 2021, with three reminders the following month.

### Statistical analyses

No sample size calculation was made beforehand since we targeted the total population of coaches within the football district. Results are presented separately for each question in the tables as well as aggregated per HAPA construct in the figures. Results are presented descriptively with median and IQR (for questions with 1—7 Likert scale) and for the constructs in the motivational phase also compared between coaches who had or had not used an IPEP (*Knee Control*, *Knee Control+* or the *11+*) using non-parametric statistics (Mann-Whitney U-test). Effect sizes, η^2^, were calculated based on Z^2^/N-1 and transformed to Cohen’s *d*. Effect sizes were interpreted as: small *d*=0.2, medium *d*=0.5 and large *d*=0.8. We also present the distribution of answers across the Likert scale to illustrate differences in responses for all HAPA constructs. Likert responses 1–2 were considered negative, 3–5 neutral and 6–7 positive. No missing data were imputed.

We studied the association between the seven HAPA constructs as well as the individual questions within each construct (independent variables) with the outcome adherence using simple logistic regression analyses. The dependent variable adherence was dichotomised into high (=coach reported use of *Knee Control+* at least two times/week) and low adherence (=used *Knee Control+* less than two times/week). This analysis was done for coaches (n=101) who had responded to all HAPA questions.

A quantitative analysis of all free-text answers on facilitators/barriers was also made, where responses were grouped together in categories and counted.

### Patient and public involvement

*Knee Control+* development was informed by a qualitative study with coaches for female teams,^7^ and pilot versions of the programme were tested and commented on by coaches and players in previous studies with male and female players.^10 18^

## Results

In total, 440 coaches (379 men, 86%, mean age 45.6 (7.4)) years, responded to the questionnaire (response rate 32%). Among these, 133 had adopted and used *Knee Control+* during the 2021 football season, and 10 had adopted and used *Knee Control+* but quit during the season (labelled users *Knee Control+*). Additionally, 144 coaches had used the IPEPs *Knee Control* or the *11+* (labelled users other IPEP). For the motivational phase of the HAPA, all 440 coaches responded, whereas only coaches who had adopted and used *Knee Control+* throughout the season (n=101 of 143, 32 coaches ended the survey prematurely) responded to questions for the goal-pursuit phase. In total, 28 coaches (6%) had attended the digital *Knee Control+* workshops during the season.

### Motivation for behaviour change

**Figure 2:**
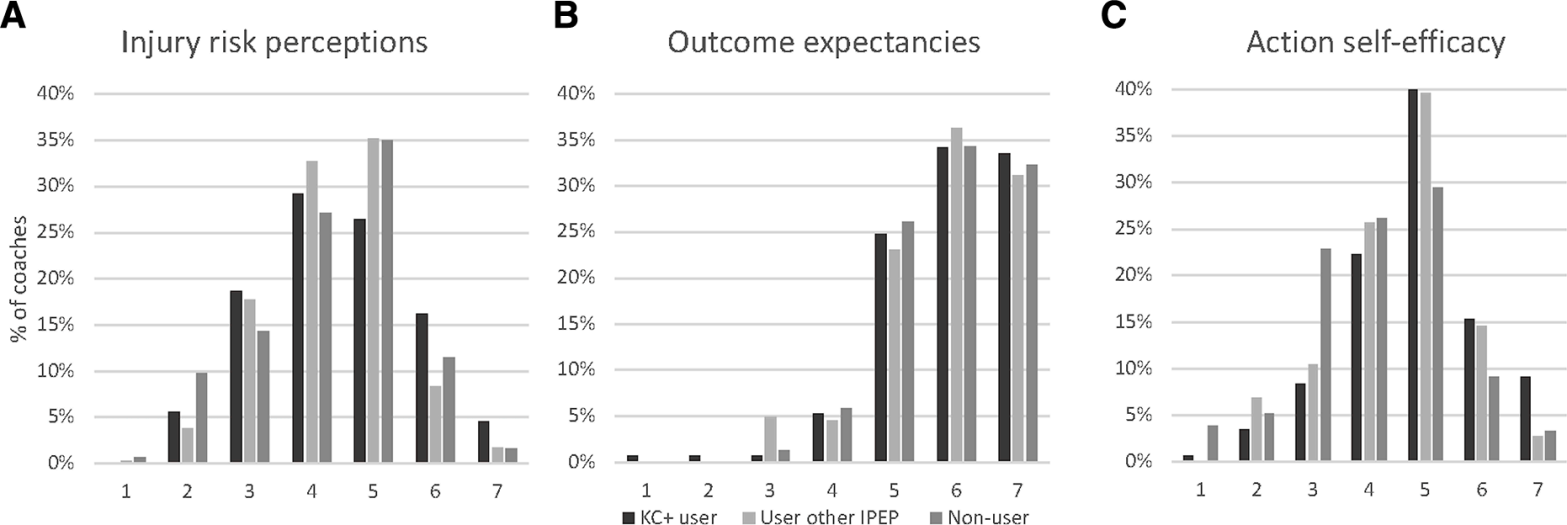
(A–C) Illustration of distribution of responses on the Likert 1–7 scale for constructs in the Health Action Process Approach (HAPA) motivational phase. 1=low, 7=high rating of the respective construct. IPEP, injury prevention exercise programme; KC+, *Knee Control+*.

Focusing on the motivational phase, coaches (n=440) were neutral (Likert 4–5) regarding injury risk perceptions, whereas they had positive outcome expectancies (Likert 6) regarding the potential injury prevention effects (table 2, figure 2A–C). As for action self-efficacy, coaches were neutral about their knowledge to prevent injuries (Likert 4–5), whereas coaches who had used *Knee Control+* were positive about their practical ability to use *Knee Control+* (Likert 6). Coaches who had used *Knee Control+* during the 2021 season also had high intention to prioritise continuous IPEP use in the subsequent season (Likert 7).

**Table 2:** Coach perceptions of injury risks, outcome expectancies and action self-efficacy

	All coaches (n=440)	Users *Knee Control+* (n=143)	Users other IPEP (n=144)	Non-users (n=153)
**Motivational phase**				
**Injury risk perceptions**				
*What do you think about the overall injury risk in football? (low–high)*	5.0 (1.0)	5.0 (2.0)	5.0 (1.0)	5.0 (1.0)
*What do you think about the injury risk in the team that you coach? (low–high)*	4.0 (2.0)*	4.0 (2.0)	4.0 (2.0)*	4.0 (2.0)
**Outcome expectancies**				
*I believe many injuries can be prevented in football (do not agree–agree)*	6.0 (1.0)*	6.0 (1.0)	6.0 (1.0)*	6.0 (1.5)
*I believe specific training can prevent injuries in football (do not agree–agree)*	6.0 (2.0)*	6.0 (1.0)	6.0 (2.0)*	6.0 (2.0)
**Action self-efficacy**				
*My knowledge about preventing injuries in football is… (inadequate–adequate)*	5.0 (1.0)	5.0 (1.0)	5.0 (1.0)	4.0 (2.0)

Health Action Process Approach constructs in bold. The respective questions are presented in italics.

Values are median (IQR). All questions are rated on a 1–7 Likert scale from 1=low/do not agree/inadequate to 7=high/agree/adequate. Groups are based on whether the respondent had used *Knee Control+* (users *Knee Control+*), *Knee Control* or the *11+* (users other IPEP) or if they had not used a complete injury prevention programme during the 2021 season (non-users). Non-users did not use a complete injury prevention programme but many used some exercises.

*1 missing answer.

IPEP, injury prevention exercise programme.

No significant differences were seen between coaches of different sexes or coaches for teams of different sexes with regards to injury risk perceptions, outcome expectancies or action self-efficacy (online supplemental table 1).

10.1136/ip-2023-044978.supp1 Supplementary data

Coaches who stated that they had used an IPEP (*Knee Control, Knee Control+* or the *11+*) during the season reported significantly higher action self-efficacy in terms of knowledge about injury prevention compared with non-users (Likert 5.0 vs 4.0, p<0.001, *d*=0.43).

### Determination for behaviour change

**Figure 3:**
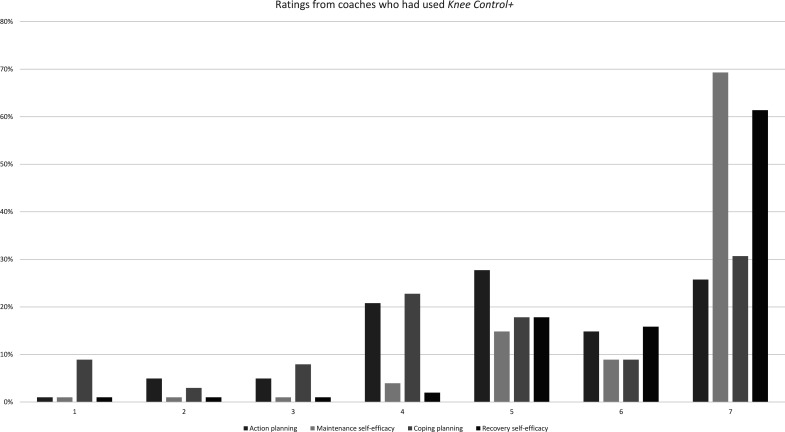
Illustration of distribution of responses on the Likert 1–7 scale for constructs in the Health Action Process Approach (HAPA) goal-pursuit phase. Likert 1=low, 7=high rating for each HAPA construct.

With regards to action and coping planning, coaches who had adopted *Knee Control+* (n=101) were neutral (Likert 5) regarding plans on how to instruct players and how to work around barriers for programme use (table 3, figure 3). As for maintenance and recovery self-efficacy, coaches fully agreed (Likert 7) that they would be able to maintain programme use over time and to start using the programme again if training was interrupted.

**Table 3:** Responses from coaches who had used *Knee Control+* during the 2021 season

	Users *Knee Control+* (n=101)
**Motivational phase**	
**Action self-efficacy**	
*My practical ability to use Knee Control+ with my team is… (inadequate–adequate)*	6.0 (2.0)
**Intention**	
*I intend to prioritise continuous Knee Control+ use in my team next season… (do not agree–agree)*	7.0 (1.0)
**Goal-pursuit phase**	
**Action planning**	
*I have concrete plans for how to instruct the players when using Knee Control+ (do not agree–agree)*	5.0 (3.0)
**Maintenance self-efficacy**	
*I believe I will be able to continue using Knee Control+ in my team next season… (do not agree–agree)*	7.0 (1.0)
**Coping planning**	
*I have plans for how to work around barriers for continued Knee Control+use… (do not agree–agree)*	5.0 (3.0)
**Recovery self-efficacy**	
*If my team stops using Knee Control+, I am certain that we can start using it again… (do not agree–agree)*	7.0 (1.0)

Health Action Process Approach constructs in bold. The respective questions are presented in italics.

Values are median (IQR). The table is based on responses from 101 coaches (out of 143 *Knee Control+*users, 10 did not receive these questions since they had stopped using *Knee Control+*, the other 32 ended the survey prematurely).

### Associations between HAPA constructs and adherence

Three HAPA questions had a statistically significant association with self-reported *Knee Control+* adherence in the simple logistic regression analyses. Increased probability of being in the high adherence group (at least 2 IPEP sessions per week) was seen with higher ratings on the questions ‘I believe specific training can prevent injuries in football’ (OR 1.64 per one-step increase on the Likert scale, 95% CI 1.04 to 2.59, p=0.034), ‘I have concrete plans for how to instruct the players when using *Knee Control+*’ (OR 1.33, 1.01 to 1.76, p=0.045), and ‘I have plans for how to work around barriers for continued *Knee Control+*use’ (OR 1.44, 1.14 to 1.81, p=0.002). No other individual questions or HAPA constructs showed an association with the outcome adherence (online supplemental table 2).

10.1136/ip-2023-044978.supp2 Supplementary data

### Facilitators and barriers for maintained use of *Knee Control+*

In total, 83 free-text responses from 43 coaches covered facilitators for programme use, and 21 barriers were described by 19 coaches. The described facilitators included: access to education in *Knee Control+* and extensive programme material (n=32 answers), time/resources such as extra time for training and having specific coaches or ‘experts’ responsible for injury prevention (n=22), availability of equipment (n=12), consensus about the importance of injury prevention between all coaches and between coaches and players (n=12), having access to digital material (n=5).

The described barriers were lack of consensus between different coaches and between coaches and players about the importance of injury prevention (n=11), lack of time and resources (n=6), lack of knowledge and equipment with difficulties realising the benefits of injury prevention (n=5) as well as external conditions such as bad weather with rain, snow, or ice on the football field and darkness during training (n=2).

## Discussion

Coaches were neutral regarding their injury risk perceptions but positive regarding the expected outcomes of injury prevention. They were neutral about action self-efficacy regarding knowledge about injury prevention training. Coaches who had used *Knee Control+* were uncertain regarding action and coping planning, whereas they described high self-efficacy to maintain programme use and to recover from interrupted training. Highly adherent coaches had higher belief in IPEP effectiveness to prevent injuries and agreed to higher extent that they had formulated action and coping plans for *Knee Control+* use.

Even though ratings suggested neutral perceptions about action self-efficacy in terms of perceived knowledge, we found a significant difference with higher ratings (Likert 5 vs 4, small effect size *d*=0.43) among coaches who used an IPEP (*Knee Control+*, *Knee Control* or the *11+*) compared with non-users. Injury risk perceptions and outcome expectancies were, however, similar between users and non-users, but the high number of neutral ratings for injury risk perceptions and action self-efficacy suggest that there is room for improvement. The median values of action self-efficacy (Likert 5 for all coaches in their perceived knowledge, Likert 6 for *Knee Control+* users in their perceived practical ability to use the programme) was similar or higher than the mean values previously reported for Canadian female adolescent football coaches evaluating the *11+* IPEP.^13^ In line with our results, action self-efficacy was highlighted as an important factor on which to focus future implementation efforts in that study, whereas injury risk information was perceived as inefficient to foster intention to adopt the IPEP.^13^ A previous qualitative study among Swedish girls’ football coaches also emphasised the importance of coach self-efficacy to enhance programme adoption and use.^7^ Offering workshops for coaches was shown as a good behaviour change strategy to improve adoption and adherence to an IPEP.^15^ Those who took part in workshops also showed higher action self-efficacy at the end of the season, suggesting that improving coach self-efficacy is one way to improve IPEP implementation.^15^ Few coaches in our study had taken part in the workshops that we offered during the season and it is unknown whether self-efficacy in using *Knee Control+* can be improved; for example, from taking part in workshops.

Focusing on the goal-pursuit phase, we found high values (Likert 7) regarding maintenance and recovery self-efficacy, suggesting that, overall, there is little room for improvement at the group level. Still, coaches may need more long-term support, such as reminders about how to use and progress the programme. Responses regarding action and coping planning were neutral at the group level (Likert 5), and coaches may need more support within these constructs to facilitate programme use. This is further emphasised by the results of the logistic regression analyses showing positive associations between adherence and the degree of action and coping planning. To help coaches to formulate plans, we could give additional suggestions in the written material and during workshops about how to integrate the programme in the usual football training and to illustrate how other coaches have successfully worked around barriers for programme use. Integrating or rescheduling IPEPs is in line with work by Whalan *et al*,^19^ who showed increased compliance when rescheduling the *11+*.

Facilitators and barriers have previously been described in surveys with a researcher-defined set of barriers.^20^ We used similar open-ended questions as McKay *et al*,^13^ and had comparable results, where access to programme material and time/resources were frequently mentioned facilitators. Lack of consensus between coaches within the team and between coaches and players about the importance of injury prevention was an important barrier. In previous studies, common barriers include programme-related factors, such as the time burden and costs, difficult exercises, or inflexible programmes, and also lack of motivation from both coaches and players.^21 22^ Another study emphasised lack of coach knowledge of the IPEP and a lack of football specificity within the programme’s content.^20^ Our study extends previous findings by emphasising the perceived importance of working towards a common goal where both coaches and players understand the importance of injury prevention. Offering education for important stakeholders such as coaches, parents and players^22^ as well as organisational leadership supporting IPEPs has been suggested to improve implementation of IPEPs.^20^ For *Knee Control+*, working to increase engagement from players and parents as well as sports organisations may be one way forward to improve coach and player buy-in and thereby use of the programme. This would be particularly valuable, considering that within the regional district where we conducted the present study, few football clubs had policies for preventive programme use.^17^ For instance, different stakeholders could be targeted with customised information material and education as well as creative and practical suggestions for how to work around the barriers that these groups report.

### Strengths and limitations

The large sample of responding coaches is a strength of the study and extends results from McKay *et al* 13 and Barden *et al*,^15^ where considerably smaller coach samples were included (n=10, n=76). Another strength was the evaluation in the applied real-world setting, since many previous studies on facilitators and barriers for IPEP implementation are imbedded in randomised trials that primarily focus on the efficacy of the IPEP.^20^ When we compared ratings in the motivational phase between coaches who had or had not used an IPEP, we found very few factors that separated these groups. This suggests that future efforts to implement IPEPs in this environment should focus less on coaches’ injury risk perceptions or outcome expectancies, and more on strengthening action self-efficacy in coaches who are unsure about how to integrate the programme in usual football training and how to perform exercises with their players.

Some limitations should be mentioned. First, we had a rather low response rate (32%), which is expected when distributing surveys outside the controlled context of randomised trials. The response rate was in line with other coach questionnaire studies.^6 23 24^ However, the risk of selection bias, with predominantly positive coaches responding, must be considered. The fact that we received responses from both users and non-users of IPEPs suggests that both positive and negative perspectives are covered. Second, when planning the workshops, we did not focus on improving specific HAPA constructs but tried to briefly cover all constructs since we did not know beforehand where support was most needed. Future implementation efforts should employ a more deliberate connection to the different HAPA constructs to facilitate behaviour change. Additionally, few of the participating coaches had attended the workshops. Third, the questionnaire was not fully validated, even though we argue that it has face validity since similar questions have been used in previous studies.^13–16^ It is unclear whether this kind of broadly scoped questionnaire with only one or two questions per construct is optimal to cover the HAPA constructs or whether other targeted approaches, such as using a validated self-efficacy scale to evaluate self-efficacy beliefs, are better suited. Additionally, these single-construct questions have fewer points of discrimination and their internal consistency cannot be determined. Furthermore, it is unknown whether a one-point difference in the Likert scale rating between groups is practically relevant. Additionally, our results are based on the coaches’ perceptions only and may not reflect whether they actually had sufficient knowledge about the programme or a good practical ability to use it. Fourth, the study was carried out in the context of Swedish amateur football, and careful consideration is needed when translating these results to other contexts.

## Conclusion

Coaches need more knowledge and support on how to use IPEPs and how to structure training to improve action self-efficacy. Coaches who had adopted *Knee Control+* had high maintenance and recovery self-efficacy but may need further support to form constructive action and coping plans how to use the programme and how to overcome potential barriers. Not only to facilitate programme use, programme material dissemination is important, but also to target initiatives towards players and other stakeholders to strengthen consensus about the importance of injury prevention.

## Data Availability

All data relevant to the study are included in the article.
