# Supplementary material for: Motivation and goal-pursuit for injury prevention training in amateur football coaches: a cross-sectional study using the Health Action Process Approach
Source: Inj Prev. 2023 Sep 11;30(1):20–6. doi: 10.1136/ip-2023-044978 (PMC10850690; doi:10.1136/ip-2023-044978)
Supplement: Supplementary data [file ip-2023-044978supp001.pdf]

Supplementary table 1. Comparisons between coaches of different sex and coaches for male versus female teams.

**Health Action Process Approach construct in bold. The respective questions are presented in italics.**	Male coaches (n =379)	Female coaches (n =61)	Comparison male vs female coaches (p-value; Cohen's *d*)	Male team (n =262)	Female team (n =163)	Comparison coaches for male vs female teams (p-value; Cohen's *d*)
**Injury risk perceptions**						
What do you think about the overall injury risk in football? (low–high)	5.0 (1.0)	5.0 (1.5)	p=0.337; *d*=0.092	5.0 (1.0)	5.0 (1.0)	p=0.359; *d*=0.089
What do you think about the injury risk in the team that you coach? (low–high)	4.0 (2.0)*	4.0 (2.0)	p=0.475; *d*=0.068	4.0 (2.0)	4.0 (2.0)*	p=0.786; *d*=0.026
**Outcome expectancies**						
I believe many injuries can be prevented in football (do not agree–agree)	6.0 (2.0)*	6.0 (1.0)	p=0.816; *d*=0.022	6.0 (1.0)	6.0 (1.0)*	p=0.325; *d*=0.095
I believe specific training can prevent injuries in football (do not agree–agree)	6.0 (1.3)*	6.0 (2.0)	p=0.377; *d*=0.084	6.0 (2.0)	6.0 (1.3)*	p=0.624: *d*=0.048
**Action self-efficacy**						
My knowledge about preventing injuries in football is… (inadequate–adequate)	5.0 (1.0)	5.0 (1.0)	p=0.329; *d*=0.093	5.0 (1.0)	5.0 (1.0)	p=0.054; *d*=0.188

Values are median (IQR), p-values and effect sizes, Cohen's *d*. All questions are rated on a 1–7 Likert scale from 1 = low/do not agree/inadequate to 7=high/agree/adequate. Responses from the 15 coaches who responded that they coached both male and female teams are not presented in the table or comparisons. All comparisons were made with Mann Whitney U-test and effect sizes eta squared were transformed to Cohen's d.

* 1 missing answer.
