# Supplementary material for: Motivation and goal-pursuit for injury prevention training in amateur football coaches: a cross-sectional study using the Health Action Process Approach
Source: Inj Prev. 2023 Sep 11;30(1):20–6. doi: 10.1136/ip-2023-044978 (PMC10850690; doi:10.1136/ip-2023-044978)
Supplement: Supplementary data [file ip-2023-044978supp002.pdf]

BMJ Publishing Group Limited (BMJ) disclaims all liability and responsibility arising from any reliance placed on this supplemental material which has been supplied by the author(s)

Supplementary Table 2. Simple logistic regression on the association between Health Action Process Approach Likert questions and self-reported adherence to the *Knee Control+* programme as dependent variable

**Health Action Process Approach construct in bold. The respective questions are presented in italics.**	Odds ratio (95% CI) for having high adherence	p-value
**Injury risk perceptions**	1.057 (0.733-1.525)	0.767
What do you think about the overall injury risk in football? (low–high)	0.916 (0.648-1.294)	0.619
What do you think about the injury risk in the team that you coach? (low–high)	1.176 (0.850-1.627)	0.328
**Outcome expectancies**	1.411 (0.895-2.225)	0.139
I believe many injuries can be prevented in football (do not agree–agree)	1.099 (0.761-1.589)	0.615
I believe specific training can prevent injuries in football (do not agree–agree)	**1.641 (1.039-2.592)**	**0.034**
**Action self-efficacy**	1.218 (0.871-1.703)	0.248
My knowledge about preventing injuries in football is… (inadequate–adequate)	1.248 (0.893-1.745)	0.195
My practical ability to use Knee Control+ with my team is… (inadequate–adequate)	1.125 (0.855-1.480)	0.402
**Action planning**		
I have concrete plans for how to instruct the players when using Knee Control+ (do not agree–agree)	**1.332 (1.007-1.762)**	**0.045**
**Maintenance self-efficacy**		
I believe I will be able to continue using Knee Control+ in my team next season… (do not agree–agree)	1.340 (0.933-1.925)	0.113
**Coping planning**		
I have plans for how to work around barriers for continued Knee Control+ use… (do not agree–-agree)	**1.435 (1.136-1.812)**	**0.002**
**Recovery self-efficacy**		
If my team stops using Knee Control+, I am certain that we can start using it again… (do not agree–agree)	1.353 (0.930-1.968)	0.114

All questions are rated on a 1–7 Likert scale from 1 = low/do not agree/inadequate to 7 = high/agree/adequate. Adherence was based on coach self-reported weekly use of *Knee Control+* and dichotomised into “high adherence” (= having used *Knee Control+* ≥ 2 times/week) and “low adherence” (= having used *Knee Control+* < 2 times/week). The analysis is based on responses from the 101 coaches who used *Knee Control+* and responded to questions from both phases of the Health Action Process Approach model. Significant results (p<0.05) are depicted in bold.
